# Elective Generator Replacement of the Subcutaneous Implantable Defibrillator—Always a Simple Pit Stop?

**DOI:** 10.1111/jce.16638

**Published:** 2025-03-20

**Authors:** Fabian Fastenrath, Sara Wuerfel, Volker Liebe, Ibrahim Akin, Daniel Duerschmied, Martin Borggrefe, Juergen Kuschyk, Boris Rudic

**Affiliations:** ^1^ Cardiology, Angiology, Haemostaseology, and Medical Intensive Care, Medical Centre Mannheim, Medical Faculty Mannheim Heidelberg University Mannheim Germany; ^2^ European Center for AngioScience (ECAS), German Center for Cardiovascular Research (DZHK) partner site Heidelberg/Mannheim, and Centre for Cardiovascular Acute Medicine Mannheim (ZKAM), Medical Centre Mannheim Heidelberg University Mannheim Germany

**Keywords:** defibrillation efficacy, defibrillation failure, generator replacement, implantable cardioverter‐defibrillator, shock impedance, S‐ICD, subcutaneous defibrillator

## Abstract

**Background and Aims:**

The safety and efficacy of the subcutaneous implantable cardioverter‐defibrillator (S‐ICD) has been proven in various clinical trials. Data on device replacement strategies is scarce. This study aims to evaluate long‐term shock efficacy, trends in shock impedance, and the effects of a replacement strategy at elective device replacement.

**Methods:**

Between January 2018 and November 2022, a total of 99 consecutive patients underwent S‐ICD device replacement. Shock impedance was determined at initial implantation, before and after device replacement. In case of high shock impedance, additional optimization of lead and generator position was performed if applicable, as well as the removal of fibrous encapsulation (“capsulectomy”) of the S‐ICD pocket.

**Results:**

Defibrillation testing with the first 65 J shock was successful in 87% of patients after initial device implantation versus 85% with the same device immediately before device replacement (*p* = 0.88). Eight patients preoperatively failed defibrillation testing with 65 J and 80 J. After a mean dwell time of 65 ± 21 months, shock impedance increased significantly from 82 ± 23 Ω at initial implantation to 98 ± 37 Ω at replacement (*p* = 0.004). Capsulectomy and optimized repositioning of the S‐ICD lead and generator resulted in a significant decrease of shock impedance to 81 ± 25 Ω as compared to 98 ± 37 Ω before the replacement (*p* = 0.004). 65 J shock efficacy improved to 95%. Postoperative defibrillation testing was successful in all patients. No acute complications were observed.

**Conclusion:**

High conversion rates at device replacement are achievable through optimized replacement strategy. In addition to improving the lead and generator position, capsulectomy appears to be a safe and effective way to reduce shock impedance and might contribute to overall system performance.

## Graphical abstract

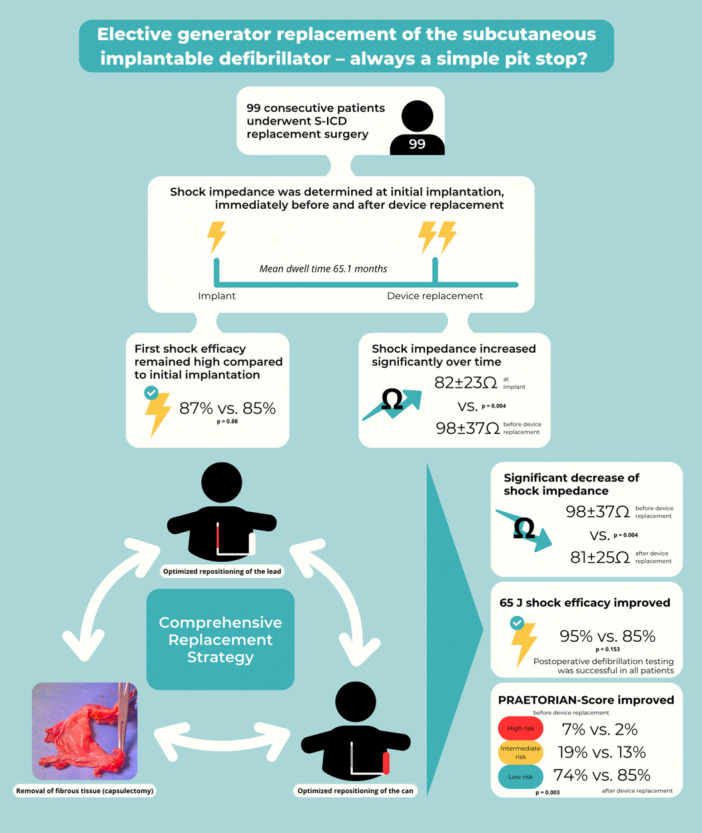

## What's New?

− Shock impedance and VF defibrillation success were obtained in S‐ICD systems with elective replacement indicator (ERI) immediately prior to generator exchange to identify patients at risk for defibrillation failure.
− Impedance increases in all S‐ICD patients with longer device dwell time and can effectively and safely be reduced by performing the described replacement strategy without increased short‐term complications.
− Shock impedance is higher in patients with VF defibrillation failure. This is likely secondary to various clinical as well as implantation‐related reasons.
− Failure to effectively terminate induced VF in S‐ICD patients demands a thorough replacement strategy, including optimization of the lead and generator position and depth. S‐ICD shock efficacy can be improved through a comprehensive replacement strategy.

## 1 Introduction

The subcutaneous implantable cardioverter‐defibrillator (S‐ICD) proved its safety and efficacy in several clinical trials [1, 2]. As experience grows, more data becomes available regarding the real‐life performance of the system [3]. This has already led to new implantation techniques and improvement of device placement in de novo implantations [4]. In contrast to transvenous ICDs, defibrillation testing at the end of every S‐ICD implantation is still recommended by the manufacturer and guidelines. Through its entirely extrathoracic placement, higher energy is required to achieve reliable shock efficacy compared to conventional transvenous ICD devices. Computer simulations, as well as real‐life data, have shown that there is a strong correlation between shock efficacy and the amount of insulating tissue between the system and the myocardium [5]. This has substantially been considered in the development of the PRAETORIAN score, which aims to predict the probability of successful defibrillation testing [6]. To evaluate whether the use of the PRAETORIAN score can replace default VF defibrillation testing in de novo implantations, the PRAETORIAN‐DFT trial was initiated. However, data on shock efficacy at the time of device replacement is limited. As observed in transvenous devices, fibrous encapsulation of the S‐ICD device occurs, and it is associated with increased dwell time in S‐ICD patients as well [7]. With growing experience with S‐ICD device replacement procedures, we and others have observed a trend toward higher shock impedance with longer device dwell time [8, 9]. In many cases, this observation was accompanied by a significant device encapsulation of the S‐ICD and/or the defibrillation lead, potentially acting as an electrical insulator [10, 11]. This study aimed to evaluate the effects of dwell time on intraoperative defibrillation testing results, the impact of optimized placement of generator and/or lead during replacement surgery, as well as the effects of device encapsulation on shock impedance as a surrogate for tissue resistance and confounder of the shock efficacy.

## 2 Methods

### 2.1 Study Population

A total of 99 consecutive patients underwent replacement of the S‐ICD generator at the University Medical Center Mannheim between January 2018 and November 2022. Demographic data, underlying comorbidities, information regarding primary implantation of the S‐ICD, as well as procedure‐related data during generator replacement were collected using medical record files. The study was approved by the institutional review board.

### 2.2 Preoperative Assessment

Available chest X‐rays were reviewed, applying the PRAETORIAN score and verification of the optimal position of the defibrillation lead and dorsal alignment of the generator. Before device replacement, shock impedance and system integrity were assessed by device interrogation. R‐wave synchronous 10 J test shock was applied to assess the shock impedance (low‐voltage shock). Then, the efficacy of the initially implanted system was tested by performing defibrillation testing. Ventricular fibrillation (VF) was induced through a 50‐Hz burst delivered by the implanted S‐ICD. VF defibrillation testing was performed in a stepwise manner, starting with 65 J. For the second S‐ICD test, we used 80 J to achieve the closest correlation to real‐world circumstances. In case of unsuccessful internal defibrillation, external defibrillation was applied. The test was declared successful if the first or second shock effectively terminated induced VF. Otherwise, the test was declared unsuccessful.

### 2.3 Intraoperative Assessment of the S‐ICD Pocket

The device replacement procedure was performed under local anesthesia and deep conscious sedation. All devices were originally placed in an intermuscular position. After the skin incision, preparation of the device pocket between the serratus anterior and latissimus dorsi muscle was performed. After the successful removal of the device, the presence of device encapsulation was visually assessed, followed by an additional dissection of the fibrous tissue (“capsulectomy”) in case of increased shock impedance (≥ 90 Ω). Additional lead revision was performed if the lead to sternum distance was > 3 coil widths. Afterward, a new S‐ICD generator was placed in the previous pocket and connected to the lead. In the case of suboptimal positioning, device placement was optimized with the implantation of the new generator. After closure of the pocket and the incision site, a test shock with 10 J and defibrillation testing with 65 J was routinely performed in all patients in the absence of contraindications for VF defibrillation testing.

### 2.4 Statistical Analysis

Descriptive statistics were used for baseline characteristics. Variables with normal distribution were presented as mean ± standard deviation (SD). Nonparametric continuous data were presented as median with interquartile range (IQR). Categorical data were presented as frequencies with percentages. For comparison of nominal values, the *χ* ^2^ and Fisher's exact tests were used, as appropriate. The Student's *t*‐test, Mann–Whitney *U* test, and Wilcoxon signed‐rank test were used for comparison of continuous or categorical variables depending on the distribution of data. *p* values ≤ 0.05 were considered significant. The SPSS software package (Chicago, Illinois, the United States; version 28) was used for analysis.

## 3 Results

### 3.1 Study Cohort

The cohort consisted of 99 consecutive patients; 77% were male, and the mean age averaged 56.1 ± 16.5 years at the time of device replacement. All patients underwent the first implantation at the University Medical Center Mannheim. 67 (68%) patients received the S‐ICD for primary prevention and 32 (32%) for secondary prevention. The underlying heart disease, along with other study cohort characteristics such as demographic data, comorbidities, left ventricular ejection fraction, and device‐related information, are displayed in Table 1. The baseline PRAETORIAN score was available and assessed in 86 of 99 patients (87%). In these patients, the PRAETORIAN score showed a low risk (score value < 90) of defibrillation failure in 64 individuals (74%), an intermediate risk (score value 90–150) of defibrillation failure in 16 (19%), and a high risk (score value > 150) of defibrillation failure in 6 (7%) individuals at initial device implant, respectively.

**Table 1:** Demographic data of the included patients.

Baseline characteristics	*n* = 99
Mean age at replacement, years (± SD)	56.1 (± 16.5)
Female, *n* (%)	23 (23)
Secondary prevention, *n* (%)	32 (32)
Underlying cardiac disease, *n* (%)	
Ischemic cardiomyopathy	28 (28)
Nonischemic cardiomyopathy	41 (41)
Channelopathies	24 (24)
Other	6 (6)
Mean LVEF at replacement, % (± SD)	42.3 (± 13.8)
Mean BMI at replacement, kg/m^2^ (± SD)	29.2 (± 7.2)
Chronic kidney disease, *n* (%)	10 (10)
Diabetes mellitus, *n* (%)	18 (18)
Arterial hypertension, *n* (%)	47 (48)
Atrial fibrillation, *n* (%)	11 (11)
S‐ICD model, *n* (%)	
1010 SQ‐RX	33 (33)
Emblem A209	63 (64)
Emblem A219	3 (3)
Median dwell time, months (IQR)	65.1 (20.7)
PRAETORIAN score at implant, *n* (%)	*missing in *n* = 13 (13)
Low risk of defibrillation failure	64 (74)
Intermediate risk of defibrillation failure	16 (19)
High risk of defibrillation failure	6 (7)

### 3.2 Preoperative VF Defibrillation Testing

At the time of device replacement, 54 (55%) patients underwent 65 J defibrillation testing preoperatively. Of the remaining 45 (45%), four had ventricular thrombi diagnosed in transesophageal echocardiography. In three patients, VF induction was omitted due to technical issues (devices affected by field safety notice regarding premature battery depletion and potential electrical shortening due to moisture ingress). In another two patients, sustained VF could not be induced despite multiple attempts. In two patients, preoperative testing was omitted due to respiratory issues. One S‐ICD pulse generator was too depleted to initiate telemetric communication. Another patient was not tested due to pregnancy. The remaining 32 patients (32%) did not undergo preoperative defibrillation testing. However, preoperative low voltage synchronous 10 J shock as a determinant of long‐term shock impedance was available in 93 patients (94%).

First shock efficacy with 65 J was 85.2% at the time of generator replacement, as compared to 87.2% at initial implantation (*p* = 0.877) (Table 2). Shock impedance increased significantly from 81.5 ± 22.9 Ω at initial implantation to 98.1 ± 37.0 Ω at the time of replacement (*p* = 0.004) and was significantly higher in patients with failed shock compared to patients with successful first shock conversion (157Ω [IQR 82] vs. 85Ω [IQR 36], *p* = 0.027). The group with unsuccessful shock had a lower fraction of “low‐risk of defibrillation failure” as well as a significantly larger fraction of “intermediate‐” and “high‐risk for defibrillation failure” regarding the PRAETORIAN score (*p* = 0.028) (Table 3). Clinical factors associated with a higher likelihood of defibrillation failure could not be identified. In the group with failed shock during 65 J defibrillation testing, mean BMI was 33.3 ± 8.6 versus 29.0 ± 6.6 kg/m^2^ (*p* = 0.11) and device dwell time was 71.2 ± 11.2 as opposed to 64.7 ± 13.0 months (*p* = 0.187) in patients with effective 65 J shock.

**Table 2:** Data comparison between first implantation versus before replacement.

	At first implantation (*N* = 88; 89%)	At replacement (*N* = 54; 55%)	*p* value
Successful first shock defibrillation (65 J), *n* (%)	77 (87%)	46 (85%)	0.877
Mean shock impedance, Ω (± SD)	81.5 (± 22.9)	98.1 (± 37)	**0.004**
Procedural time (min), mean (± SD)	50 (± 18)	42 (± 24)	**0.011**
Ejection fraction, % (± SD)	40.0 (± 18)	42.3 (± 14)	0.349
Mean BMI, kg/m^2^ (± SD)	26.7 (± 8.6)	28.6 (± 8.3)	0.196

*Note:* Bold values indicate statistically significant at *p* < 0.05.

**Table 3:** Data comparison between patients with successful and unsuccessful VF defibrillation testing before replacement.

	Successful VF defibrillation *n* = 46		Unsuccessful VF defibrillation *n* = 8		*p* value
Median shock impedance, Ω (IQR)	85 (35)		140 (76)		**0.01**
Ejection fraction, % (± SD)	42.2 (± 14.6)		42 (± 8.1)		0.975
Mean BMI, kg/m^2^ (± SD)	29 (± 6.6)		33.3 (± 8.6)		0.111
PRAETORIAN score, *n* (%)	*missing in *n* = 4		*missing in *n* = 2		
Low risk of defibrillation failure	29 (69)		1 (17)		**0.028**
Intermediate risk of defibrillation failure	8 (19)		4 (67)		
High risk of defibrillation failure	5 (12)		1 (17)		
Mean dwell time, months (± SD)	64.7 (± 13.0)		71.2 (± 11.2)		0.187
Effective VF defibrillation with first 65 J shock before replacement, *n* (%)	46 (100)	*p* = 1.0	0 (0)	*p* = **0.016**	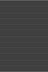
Effective VF defibrillation with first 65 J shock after replacement, *n* (%)	45 (98)		7 (88)		

*Note:* Bold values indicate statistically significant at *p* < 0.05.

In the group of patients who failed the preoperative defibrillation test, one patient had a low risk of defibrillation failure (PRAETORIAN score 30). In this specific case, the ineffective defibrillation was most likely due to the device failure as described in Boston Scientific's safety advisory issued in December 2020 (event of electrical overstress). In this patient, the S‐ICD did not effectively terminate induced VF. Moreover, after the internal shock, the device lost all telemetry functions and could not perform further interrogation. Thereby, we assume technical reasons for ineffective defibrillation.

### 3.3 Replacement Surgery and Postoperative Defibrillation Testing

**Figure 1:**
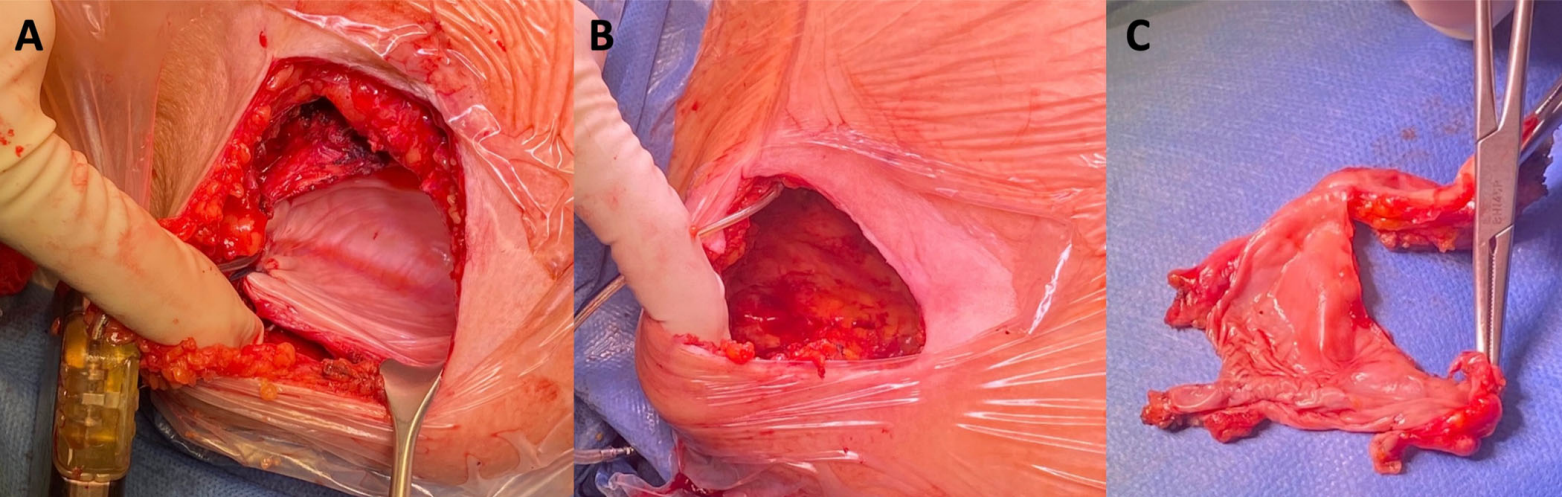
Representative example of an intraoperative preparation (A), dissection (B), and in toto removal (C) of the fibrous tissue surrounding the S‐ICD device.

**Figure 2:**
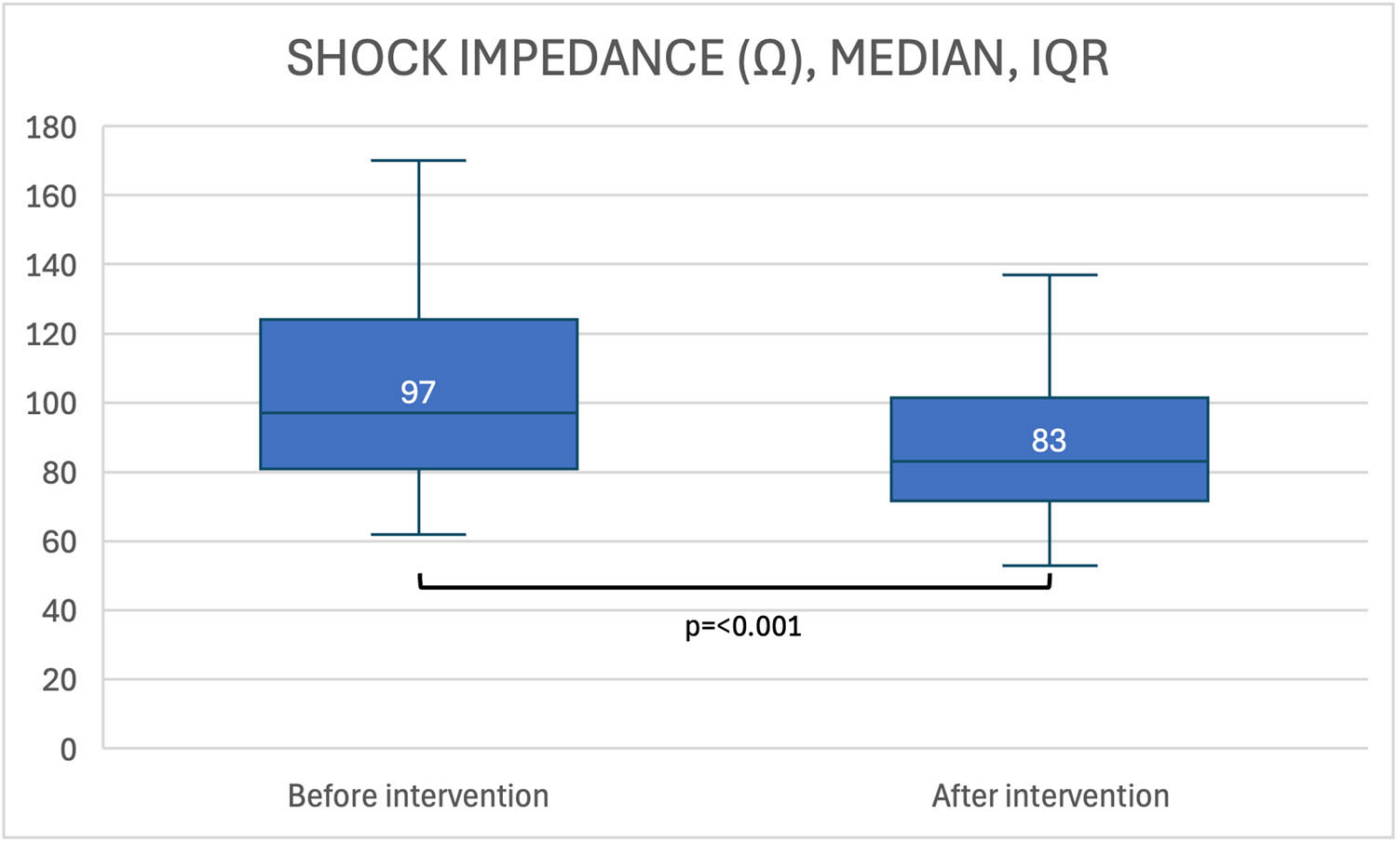
Shock impedance of 10 J (LV) testing before and after decapsulation during the replacement procedure.

**Figure 3:**
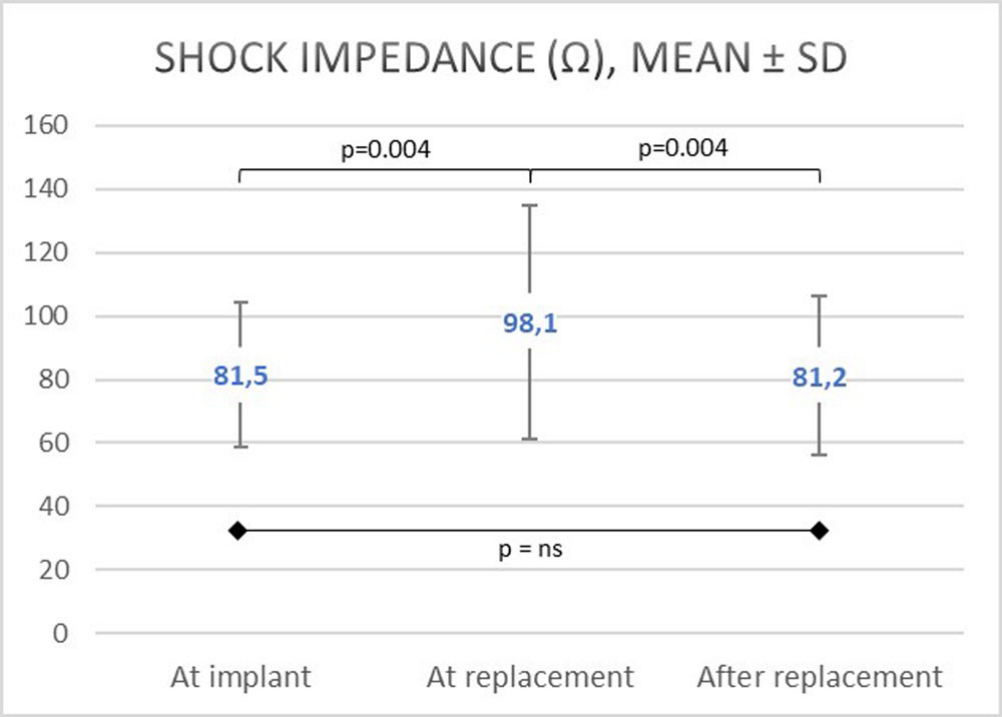
Visualization of the trend of impedance at the time of implantation, pre‐replacement, and after replacement.

Capsulectomy (Figure 1) was performed in 77 patients (78%) in case of preoperatively increased shock impedance ≥ 90 Ω or unsuccessful VF defibrillation testing. Adaptation of the lead depth closer to the sternum was necessary in 19 cases (19%), in 8 patients combined with capsulectomy and in 5 more patients combined with optimization of generator placement with an entirely new pocket being formed. In total, 6 patients were subject to generator relocation. Following device replacement, defibrillation testing with 65 J in final configuration was performed in 85 (86%) patients. As in preoperative testing, reasons for omitting defibrillation testing were the presence of ventricular thrombus or interrupted anticoagulation in persistent or permanent atrial fibrillation in five cases (5.1%), failure of sustained VF despite induction in multiple attempts in four patients (4.0%), and respiratory concerns and pregnancy in one patient, respectively. In three patients, the reason for the omission of defibrillation testing remained unknown. In patients with contraindications for defibrillation testing, a low‐voltage shock as a surrogate for the high‐voltage shock was obtained. In 30 patients with available data before and after capsulectomy, a significant decrease of shock impedance following capsulectomy was evidenced (97 Ω [IQR 43] vs. 83 Ω [IQR 30], *p* < 0.001) (Figure 2). All postoperatively tested patients had successful VF termination, with improved first shock efficacy at 65 J of 96.3% (*p* = 0.07). In two patients, the first 65 J shock failed to defibrillate induced VF. However, the subsequent 80 J shock effectively defibrillated VF in all cases. Compared to preoperative assessment, shock impedance was significantly lowered from 98.1 ± 37.0 Ω to 81.2 ± 25 Ω (*p* = 0.004) after device replacement (Figure 3). Regarding the PRAETORIAN score, the group with “low risk of defibrillation failure” increased from 74% to 85%, whereas the “intermediate risk‐” and “high‐risk for defibrillation testing failure” groups were reduced from 19% to 13% and 7% to 2%, respectively (*p* = 0.003) (Table 4). There was a trend toward lower BMI (39.8 ± 14.6 vs. 29.0 ± 6.5 kg/m^2^, *p* = 0.234) as well as a trend toward lower shock impedance in patients with effective 65 J shock (97 Ω [IQR 36] vs. 74 Ω [IQR 23], *p* = 0.075).

**Table 4:** Comparison before and after device replacement.

	Before replacement	After replacement	*p* value
Number of patients undergoing defibrillation testing	54 (55%)	85 (86%)	
Successful first shock defibrillation (65 J), %	85	96	0.07
Mean shock impedance, Ω (± SD)	98.1 (± 37)	81.2 (± 25)	**0.004**
PRAETORIAN score, *n* (%)	*missing in *n* = 13	*missing in *n* = 31	
Low risk of defibrillation failure	64 (74)	58 (85)	**0.003**
Intermediate risk of defibrillation failure	16 (19)	9 (13)	
High risk of defibrillation failure	6 (7)	1 (2)	

*Note:* Bold values indicate statistically significant at *p* < 0.05.

**Figure 4:**
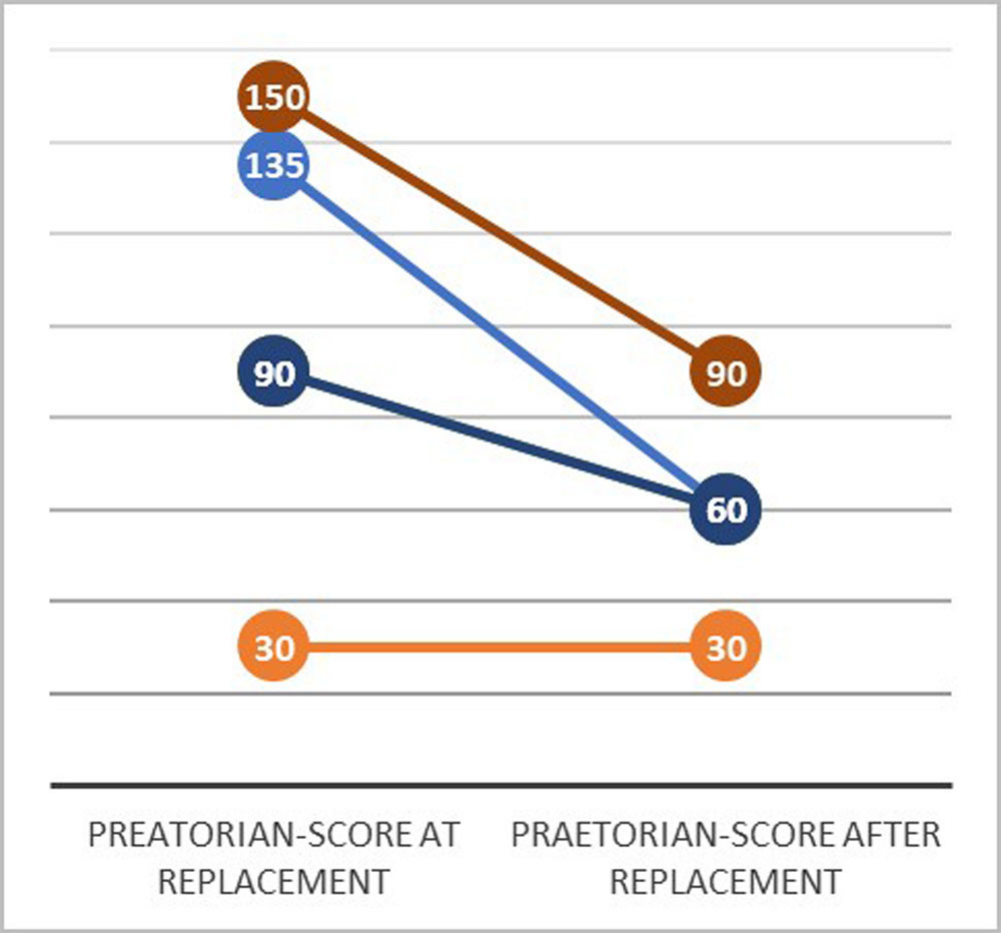
Change of the PRAETORIAN score at the time of replacement and after the optimization of lead and/or device position in the eight patients with failed VF defibrillation at replacement.

Of the eight patients with unsuccessful preoperative VF defibrillation testing, the fibrous capsule surrounding the S‐ICD generator was removed in all cases. Five (63%) were subject to lead revision, and six patients had the generator relocated (five with additional lead revision and one had generator relocation only). Shock impedance in this group was reduced from 140 Ω (IQR 76) to 76 Ω (IQR 29) (*p* = 0.008). The change in the PRAETORIAN score is visualized in Figure 4. After device replacement, VF defibrillation testing with 65 J was successful in 7/8 (88%) patients in this group and in 8/8 patients with the second 80 J shock.

The procedure time for device replacement, including previously described steps, averaged 42 ± 24 min and was significantly shorter than the procedural time of the initial de novo implantation (50 ± 18 min, *p* = 0.011). No complications (defined as significant bleeding, hematoma, prolongation of hospital stay, or wound infection) were observed.

## 4 Discussion

In this study, we report the outcome of VF defibrillation testing with prolonged dwell time of the S‐ICD system, as well as the effects of replacement strategies in a monocentric cohort. Although shock efficacy remained high and comparable to the initial implantation, we observed a significant increase in shock impedance that could effectively be reduced to baseline levels with the described replacement strategy. While the postoperative PRAETORIAN score, as well as shock effectiveness, improved, the clinical benefit of the capsulectomy still remains unknown.

### 4.1 Characteristics of the Study Cohort and Implanted Devices

There was a high number of patients with a secondary prevention indication for ICD therapy, explaining the high mean ejection fraction at the implant as well as at the time of device replacement. Furthermore, there was a high proportion of nonischemic cardiomyopathies and channelopathies present in this cohort. This is likely due to the preferred implantation of the S‐ICD in younger patients without the need for cardiac pacing. As patients usually require lifelong ICD therapy, young patients derive the biggest advantage of the S‐ICD: the entirely extrathoracic placement, abolishing the risks of lead extraction in case of endocarditis or lead failures as in transvenous ICDs [2].

The most frequently used device was the Emblem A209, which has been implanted since 2015. As there were several patients in this cohort who were initially implanted with an S‐ICD before 2015, there is a fair amount of 1010 SQ‐RX devices in our study. However, no differences in encapsulation or shock impedance trends were observed. With a median dwell time of 65 months, the proposed battery life of approximately 5 years was surpassed. Throughout this time, there was a slight, yet not statistically significant, increase in ejection fraction and BMI in the observed group.

### 4.2 Trends in Shock Impedance

Throughout the median dwell time of 65.1 months, a significant increase in shock impedance was observed. This was previously reported by our center as well as others [8, 9]. According to Amin et al. [12], the shock efficacy of the S‐ICD is significantly associated with high‐voltage impedance and device positioning. With 65 J, defibrillation efficacy was 95% with high‐voltage impedance ≤ 89 Ω, whereas the efficacy decreased to 77% with impedance ≥ 90 Ω. Through preoperative low‐voltage shocks and, in most cases, even defibrillation testing, we aimed to identify these patients at risk for defibrillation failure. Previous studies have indicated that the amount of sub‐generator fat tissue, as well as the coil–sternum distance, can significantly influence shock effectivity [5, 6, 12]. Thick fibrous tissue around the S‐ICD device was present in almost all patients in the present cohort, possibly acting as an additional isolating barrier. Additional measures during the device replacement procedure significantly lowered shock impedance. Capsulectomy was performed in most patients. Although device relocation was explicitly performed in 6/8 patients with failed first shock conversion, it can be assumed that capsulectomy resulted in slight changes to device positioning as well. Mean postoperative impedance values after replacement were comparable to shock impedance at the initial implantation. It remains unknown whether the observed increase of shock impedance increments over time or reaches a time‐dependent saturation. Further long‐term data are needed to address this question, as well as the association between shock impedance and shock effectiveness.

### 4.3 Role of Defibrillation Testing on Overall System Performance

Various trials have shown that S‐ICD shock efficacy is highly dependable on implantation technique and device placement [6, 13]. Success in defibrillation testing has been reported to be over 90% in large S‐ICD trials [1, 14]. To ensure proper system functionality before replacement, most patients underwent preoperative defibrillation testing. There was no statistically significant difference in first shock efficacy with 65 J at replacement as compared to initial implantation. Safe system performance after a median dwell time of 65 months was noted in this cohort. Although not statistically significant, first shock efficacy improved noticeably after successful device replacement. This effect is presumably influenced by several factors. Aside from the increase of the shock impedance, the learning curve effects on S‐ICD interventions have been proven to be a factor for reliable system performance [15]. As described by de Veld et al. [16], device replacement procedures offer an opportunity to revise the positioning of the system, as occurred in 75% of patients with failed preoperative defibrillation testing in this cohort. Therefore, there was a significant improvement in the PRAETORIAN score through the replacement procedure. The positive predictive value of the PRAETORIAN score is 51% in patients meeting the criteria for “high‐risk” for defibrillation failure [6]. By reducing the number of patients in this group, improved results in defibrillation testing can be expected. Nonetheless, the probabilistic nature of defibrillation testing must always be considered regarding the results of defibrillation testing [17].

### 4.4 Need for a Replacement Strategy?

With growing experience, an increasing number of centers have already started to omit defibrillation testing at initial implantation [18]. A subanalysis study of the PRAETORIAN‐DFT trial demonstrated a negative predictive value of 99% for successful DFT in patients with a PRAETORIAN score of < 90 [19]. Results from the PRAETORIAN‐DFT trial are eagerly awaited, possibly paving the way to the omission of defibrillation testing at initial S‐ICD implantation. Interestingly, in the subanalysis study, an impedance of > 100 Ω at the first DFT was an independent predictor of DFT failure.

Still regarded as a relatively new therapy (FDA approval 2012), device replacement procedures are on the rise due to the proposed battery life of ~5 years. Data on the optimal device replacement strategy are scarce. In this study, patients with failed first shock had higher shock impedance values than patients with effective defibrillation testing. By performing our replacement strategy, a significant reduction of the shock impedance was achieved. This finding was not accompanied by adverse events and prolonged surgery duration despite additional measures. Capsulectomy and, if necessary, device or lead relocation may be considered as a countermeasure for increasing shock impedance. Prospective, randomized studies are warranted to further investigate the prognostic effects of this replacement strategy.

### 4.5 Limitations

This study included a monocentric cohort of patients implanted from 2010 onward. Learning curve and implantation improvements could have influenced the outcome and the presented results. However, similar rates of first shock efficacy with 65 J at the time of initial implantation compared with generator replacement were observed, which speaks in favor of the probabilistic nature of defibrillation testing. Not all 99 patients underwent preoperative defibrillation testing. However, low‐voltage impedance was available in the majority of cases. It is noteworthy that all tested patients ultimately passed the 80 J shock test, including patients with preoperatively failed VF defibrillation tests and subsequent device/lead optimization. It remains unclear to what extent each factor of our replacement strategy (device placement, lead‐revision, and capsulectomy) contributes to the improved results after device replacement. Factors associated with the surgery itself, such as wound bed moisture, could also have influenced the results.

Due to the retrospective design and small patient cohort, we only pointed out observed trends. The clinical significance would have to be further assessed in larger study cohorts favoring a prospective study design with a matched control group.

Also, the obtained results cannot be extrapolated to spontaneous ventricular tachyarrhythmic events. However, the omission of defibrillation testing precludes the identification of patients at risk of defibrillation failure.

## 5 Conclusion

First shock efficacy of the S‐ICD remains high, even years after implantation. In patients with failed VF defibrillation testing, shock efficacy can further be improved by optimal positioning of the device and/or defibrillation lead. A significant increase in shock impedance occurs over the course of time and can effectively be reduced by a diligent replacement strategy that establishes an optimal system position. In line with the findings of the subanalysis of the PRAETORIAN‐DFT trial, where high impedance was found to be an independent risk factor for DFT failure, we showed that removal of fibrous tissue could be safely performed and might be considered in patients in whom no further optimization of PRAETORIAN score associated surgical steps are possible.

## Conflicts of Interest

J.K. has received personal fees and grant support from Boston Scientific outside of the submitted work. All other authors have reported that they have no relationships relevant to the contents of this article to disclose. The manuscript is original, with no portion under simultaneous consideration for publication elsewhere or previously published. All authors are responsible for the contents and have read and approved the manuscript for submission.

## Data Availability

The data that support the findings of this study are available on request from the corresponding author.

## References

[1] L. Boersma , C. Barr , R. Knops , et al., “Implant and Midterm Outcomes of the Subcutaneous Implantable Cardioverter‐Defibrillator Registry,” Journal of the American College of Cardiology 70, no. 7 (August 2017): 830–841, 10.1016/j.jacc.2017.06.040. 28797351

[2] R. E. Knops , L. R. A. Olde Nordkamp , P. P. H. M. Delnoy , et al., “Subcutaneous or Transvenous Defibrillator Therapy,” New England Journal of Medicine 383, no. 6 (August 2020): 526–536, 10.1056/NEJMoa1915932. 32757521

[3] A. F. B. E. Quast , V. F. van Dijk , S. C. Yap , et al., “Six‐Year Follow‐Up of the Initial Dutch Subcutaneous Implantable Cardioverter‐Defibrillator Cohort: Long‐Term Complications, Replacements, and Battery Longevity,” Journal of Cardiovascular Electrophysiology 29, no. 7 (July 2018): 1010–1016, 10.1111/jce.13498. 29626366

[4] R. E. Knops , L. R. A. Olde Nordkamp , J. R. de Groot , and A. A. M. Wilde , “Two‐Incision Technique for Implantation of the Subcutaneous Implantable Cardioverter‐Defibrillator,” Heart Rhythm 10, no. 8 (2013): 1240–1243, 10.1016/j.hrthm.2013.05.016. 23707489

[5] E. K. Heist , A. Belalcazar , W. Stahl , T. F. Brouwer , and R. E. Knops , “Determinants of Subcutaneous Implantable Cardioverter‐Defibrillator Efficacy,” JACC: Clinical Electrophysiology 3, no. 4 (2017): 405–414, 10.1016/j.jacep.2016.10.016. 29759454

[6] A. F. B. E. Quast , S. W. E. Baalman , T. F. Brouwer , et al., “A Novel Tool to Evaluate the Implant Position and Predict Defibrillation Success of the Subcutaneous Implantable Cardioverter‐Defibrillator: The PRAETORIAN Score,” Heart Rhythm 16, no. 3 (2019): 403–410, 10.1016/j.hrthm.2018.09.029. 30292861

[7] J. M. Li , Y. Li , V. Tholakanahalli , and D. G. Benditt , “Fibrous Encapsulation of Defibrillation Electrode and Elevated High‐Voltage Impedance in Patients With a Subcutaneous Implantable Cardioverter‐Defibrillator,” HeartRhythm Case Reports 6, no. 3 (March 2020): 148–152, 10.1016/j.hrcr.2019.11.009. 32181134 PMC7064802

[8] B. Rudic , E. Tülümen , F. Fastenrath , I. Akin , M. Borggrefe , and J. Kuschyk , “Defibrillation Failure in Patients Undergoing Replacement of Subcutaneous Defibrillator Pulse Generator,” Heart Rhythm Society 17, no. 3 (2020): 455–459, 10.1016/j.hrthm.2019.10.024. 31669771

[9] W. van der Stuijt , A. F. B. E. Quast , S. W. E. Baalman , et al., “Complications Related to Elective Generator Replacement of the Subcutaneous Implantable Defibrillator,” EP Europace 23, no. 3 (March 2021): 395–399, 10.1093/europace/euaa263. PMC7947576 33197266

[10] S. Jacob , V. Pidlaoan , J. Singh , A. Bharadwaj , M. B. Patel , and A. Carrillo , “High Defibrillation Threshold: The Science, Signs and Solutions,” Indian Pacing and Electrophysiology Journal 10, no. 1 (January 2010): 21–39. 20084193 PMC2803603

[11] A. Tzukert , E. Leviner , Y. Mahler , and S. Shoshan , “Electroconductivity of Collagen In Vitro and In Vivo,” Biochimica et Biophysica Acta (BBA)—General Subjects 627, no. 3 (February 1980): 276–280, 10.1016/0304-4165(80)90458-4. 7353058

[12] A. K. Amin , M. R. Gold , M. C. Burke , et al., “Factors Associated With High‐Voltage Impedance and Subcutaneous Implantable Defibrillator Ventricular Fibrillation Conversion Success,” Circulation: Arrhythmia and Electrophysiology 12, no. 4 (April 2019): e006665, 10.1161/CIRCEP.118.006665. 30917689

[13] P. Francia , C. Adduci , A. Angeletti , et al., “Acute Shock Efficacy of the Subcutaneous Implantable Cardioverter‐Defibrillator According to the Implantation Technique,” Journal of Cardiovascular Electrophysiology 32, no. 6 (June 2021): 1695–1703, 10.1111/jce.15081. 33969578

[14] L. V. Boersma , M. F. El‐Chami , M. G. Bongiorni , et al., “Understanding Outcomes With the Emblem S‐ICD in Primary Prevention Patients With Low EF Study (UNTOUCHED): Clinical Characteristics and Perioperative Results,” Heart Rhythm 16, no. 11 (2019): 1636–1644, 10.1016/j.hrthm.2019.04.048. 31082539

[15] R. E. Knops , T. F. Brouwer , C. S. Barr , et al., “The Learning Curve Associated With the Introduction of the Subcutaneous Implantable Defibrillator,” EP Europace 18, no. 7 (July 2016): 1010–1015, 10.1093/europace/euv299. PMC4927061 26324840

[16] J. A. de Veld , S. Pepplinkhuizen , W. van der Stuijt , et al., “Successful Defibrillation Testing in Patients Undergoing Elective Subcutaneous Implantable Cardioverter‐Defibrillator Generator Replacement,” EP Europace 25, no. 7 (July 2023): euad184, 10.1093/europace/euad184. PMC10325005 37379530

[17] J. Hayase , D. H. Do , and N. G. Boyle , “Defibrillation Threshold Testing: Current Status,” Arrhythmia & Electrophysiology Review 7, no. 4 (December 2018): 288–293, 10.15420/aer.2018.54.2. 30588318 PMC6304797

[18] D. J. Friedman , C. S. Parzynski , P. D. Varosy , et al., “Trends and In‐Hospital Outcomes Associated With Adoption of the Subcutaneous Implantable Cardioverter Defibrillator in the United States,” JAMA Cardiology 1, no. 8 (November 2016): 900–911, 10.1001/jamacardio.2016.2782. 27603935 PMC5112106

[19] R. E. Knops , M. F. El‐Chami , C. Marquie , et al., “Predictive Value of the PRAETORIAN Score for Defibrillation Test Success in Patients With Subcutaneous ICD: A Subanalysis of the PRAETORIAN‐DFT Trial,” Heart Rhythm 21, no. 6 (June 2024): 836–844, 10.1016/j.hrthm.2024.02.005. 38336193

